# Improvement in early continence after introduction of periurethral suspension stitch in robotic prostatectomy

**DOI:** 10.1007/s11701-020-01156-6

**Published:** 2020-10-14

**Authors:** Erling Aarsæther, Marius Roaldsen, Tore Knutsen, Hiten R. Patel, Bård Soltun

**Affiliations:** 1 Department of Urology, University Hospital of North Norway, 9038 Tromsø, Norway; 2 Department of Clinical Medicine, UiT-Arctic University of Norway, Tromsø, Norway

**Keywords:** Prostate cancer, Robotic prostatectomy, Suspension stitch, Urinary continence

## Abstract

Early urinary incontinence remains a major source of morbidity for patients undergoing robotic prostatectomy. The purpose of the study was to determine whether the introduction of a suspension stitch would improve early urinary continence rates in patients undergoing robotic prostatectomy for localized prostate cancer at our department. We retrospectively reviewed patients undergoing robotic prostatectomy with either suspension (*n* = 119) or figure-of-eight (*n* = 48) stitching of the dorsal venous complex. The patients submitted EPIC-26 questionnaires before surgery and after 3 and 18 months, respectively. Logistic regression analysis was run to determine the effect of the suspension stitch, nerve-sparing, posterior reconstruction, prostate volume, age and body mass index on early continence rate. The odds ratio of experiencing urinary leaks was 2.1 times higher (95% CI 1.0–4.3) in the figure-of-eight stitch group compared to the suspension stitch group 3 months after surgery (*p* < 0.05). The early urinary continence rate was 61.3% in the suspension stitch group compared to 35.4% in the figure-of-eight stitch group (*p* < 0.005). There were no differences between the groups 18 months post-prostatectomy (90.7% in the suspension stitch group versus 81.4% in the non-suspension stitch group, *p* = 0.1). Ordinal regression analysis identified the suspension stitch, bilateral nerve-sparing and body mass index as independent predictors of urinary continence at 3 months. The association between urinary continence and either unilateral nerve-sparing, posterior reconstruction, prostate volume or age did not reach statistical significance. Our results suggest that the suspension stitch improved early urinary continence following robotic prostatectomy.

**Electronic supplementary material:**

The online version of this article (10.1007/s11701-020-01156-6) contains supplementary material, which is available to authorized users.

## Introduction

Robotic prostatectomy is currently an established treatment option for localized prostate cancer, providing similar oncological outcomes to open surgery [1, 2] and demonstrating favorable results on transfusion rates and length of hospital stay [3, 4]. Urinary continence rates from large robotic centers are now exceeding 90% after 1 year [5]. However, early incontinence remains an area in which there is a potential for improvement, as results are still substantially worse than those of the long term [5]. Technical refinements to the surgical procedure, such as posterior reconstruction of the rhabdosphincter and nerve-sparing, have improved results [6, 7]. However, given the substantial number of patients who are subjected to robotic prostatectomy each year, short-term incontinence remains a major source of morbidity. The purpose of the study was to determine whether the introduction of the retropubic suspension stitch would improve short-term urinary continence in patients undergoing robotic prostatectomy for localized prostate cancer at our department.

## Methods

The study was approved by the regional ethical committee, and informed written consent was obtained from all participants. Two hundred and ten patients who underwent robotic prostatectomy at the University Hospital of North Norway between May 28, 2015, and July 13, 2017, with or without the retropubic suspension stitch were prospectively enrolled in the study. In this particular period of time, all the four surgeons in the robotic program gradually changed their operating technique from the previously established figure-of-eight stitch to the retropubic suspension stitching technique. The four surgeons involved had completed at least 100 cases each, with one having previous experience from robotic centers in the UK and USA.

All patients were asked to answer the expanded prostate cancer index composite, EPIC-26, before surgery, and after 3 and 18 months, respectively. Patient forms were submitted anonymously to the department by mail. Clinical and quality-of-life-related data were prospectively scanned and registered in a customized database. Patients who failed to submit EPIC-26 forms 3 months post-prostatectomy were excluded from the study. Missing quality-of-life data at 18 months were supplemented by making individual phone calls. All data were retrospectively analyzed and reviewed. Between-group differences were analyzed with the Student’s *t* test for numerical data and the chi-square test for categorical data. Likert scale data from the EPIC-26 forms were analyzed using a cumulative odds ordinal logistic regression with proportional odds to determine the effect of the suspension stitch, nerve-sparing, posterior reconstruction, prostate volume, age and body mass index on the early urinary continence rate. All statistical analyses were performed with the IBM SPSS Statistics 24.

### Surgical technique

**Fig. 1:**
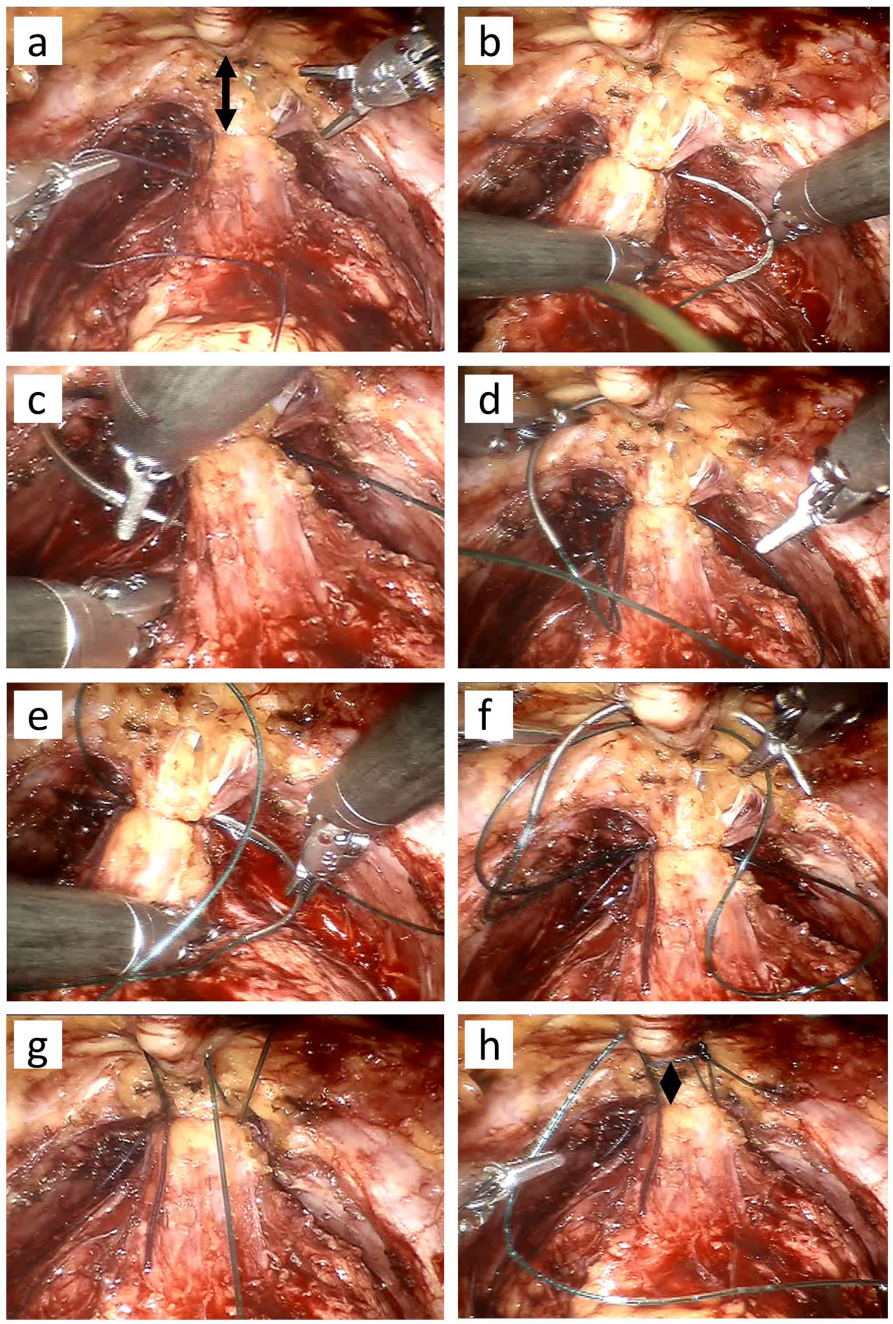
**a**–**h** The periurethral retropubic suspension stitch. The DVC was first ligated with a hemostatic suture. The periurethral retropubic suspension stitch was passed from right to left between the DVC and the urethra and then through the perichondrium of the pubic bone twice. Arrows in image (**a**) and (**h**) indicate how the distance between the hemostatic suture decreases after the suspension suture is tied

**Fig. 2:**
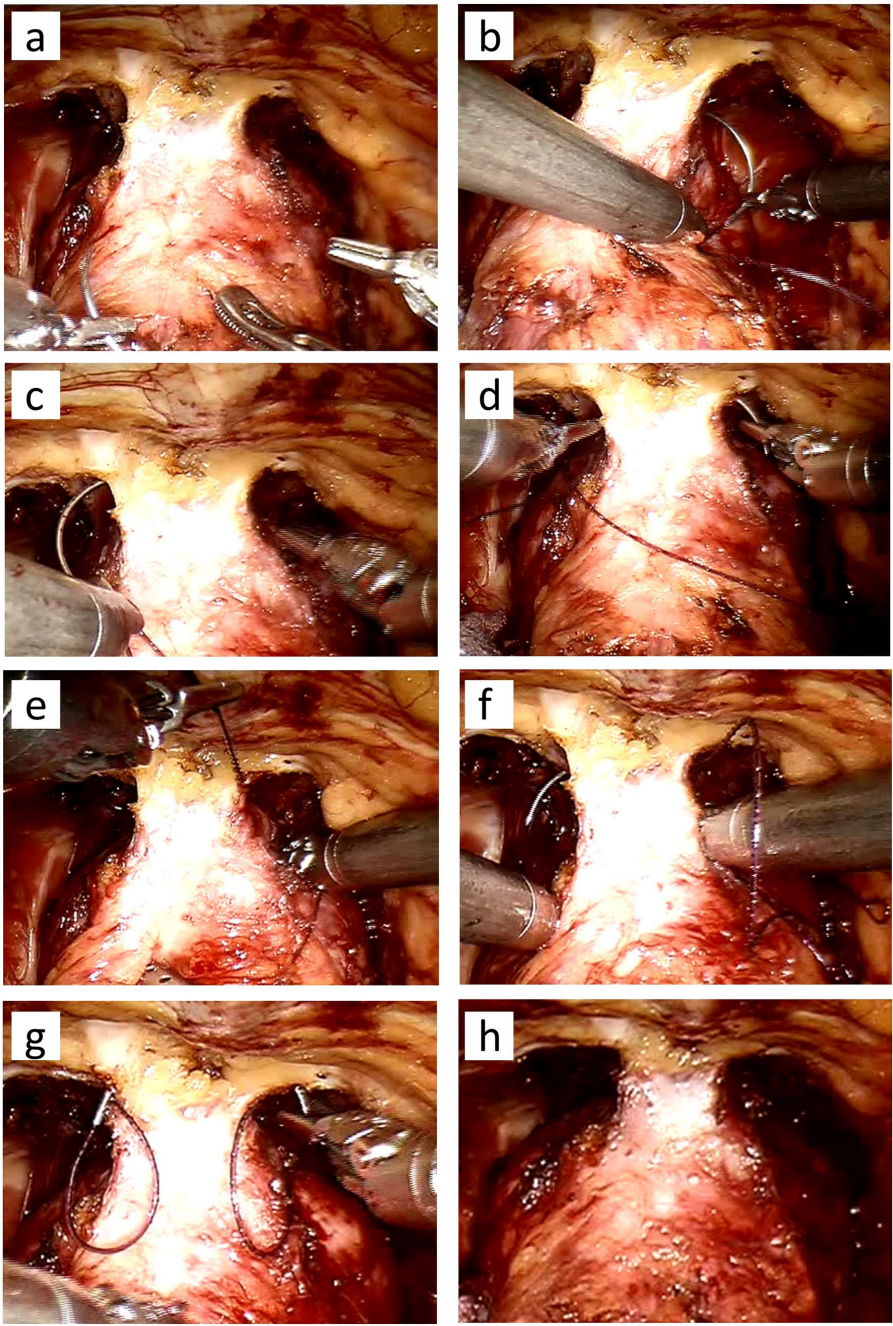
**a**–**h** The figure-of-eight stitch. In the non-suspension group, the DVC was ligated with an absorbable suture from right to left in a figure of eight

All patients underwent robotic prostatectomy in a standardized procedure with the four-arm da Vinci robot (Intuitive Surgical, Sunnyvale, Cal., USA) and an additional two 10-mm trocars for laparoscopic assistance. The two groups were retrospectively separated by which of the two stitching procedures that were utilized on the dorsal venous complex (DVC). In the retropubic suspension stitch group, the DVC was first ligated with an absorbable suture (Polysorb 2–0, Covidien, Dublin, Ireland). Following the ligation of the DVC, a periurethral retropubic stitch (Maxon 2–0, Covidien) was passed from right to left between the DVC and the urethra and then through the perichondrium of the pubic bone twice (Fig. 1a–h), as previously described [8]. In the non-suspension group, the DVC was ligated with our standard method, utilizing an absorbable suture from right to left in a figure of eight (Fig. 2a–h).

### Urinary continence

Questions from the EPIC-26 form utilized to describe continence in this study were the following: Over the past four weeks, how often have you leaked urine?, and how many pads or adult diapers per day did you usually use to control urine leaking the last 4 weeks?. Urinary continence was defined as no pad or a safety pad.

## Results

EPIC-26 forms were obtained from 167 of 210 patients (79.5%) 3 months post-robotic prostatectomy. Of these 167 patients, 119 (71%) underwent robotic prostatectomy with the retropubic suspension stitch, whereas 48 patients (29%) underwent robotic prostatectomy with a standard figure-of-eight DVC stitch (Figs. 1, 2a–j). There were no differences between the groups with respect to age, body mass index, preoperative PSA, ASA score or prostate volume (Table 1). The number of patients who underwent robotic prostatectomy with bilateral nerve-sparing was similar in the two groups (Table 2). However, patients in the non-suspension group were more likely to undergo surgery without nerve-sparing compared to patients in the retropubic suspension stitch group. Mean blood loss and the number of patients who had robotic prostatectomy without pelvic lymph node dissection were higher in the non-suspension group than in the suspension group, but the difference was not significant at the 5% level. There was no difference in operative time between the groups, and the catheter time was almost identical (Table 2).

**Table 1:** Patient characteristics

Patient characteristics	Without suspension (*n* = 48)	With suspension (*n* = 119)	*p*
Age, mean ± SD	65.4 ± 5.3	64.1 ± 6.5	0.2
Body mass index, kg/m^2^, mean ± SD	26.5 ± 3.1	26.9 ± 3.5	0.4
PSA before RALP, µg/L, mean ± SD	10.7 ± 10.7	9.9 ± 5.8	0.5
ASA score = 2, (%)	42 (87.5%)	100 (84.0%)	0.6
ASA score = 3, (%)	6 (12.5%)	19 (16%)	0.6
Prostate volume (g), mean ± SD	50.6 ± 15	48.6 ± 18	0.5

*ASA* American Society of Anaesthesiologists, *PSA* prostate-specific antigen, *RALP* robotic-assisted laparoscopic prostatectomy, *SD* standard deviation

**Table 2:** Perioperative parameters

Perioperative parameters	Without suspension (*n* = 48)	With suspension (*n* = 119)	*p*
Nerve-sparing procedure, no. (%)			
Bilateral	9/48 (18.8%)	26/119 (21.8%)	0.7
Unilateral	17/48 (35.4%)	59/119 (49.6%)	0.1
None	22/48 (45.8%)	34/119 (28.6%)	< 0.05
Posterior reconstruction, no. (%)	14 (25.0%)	30 (26.1%)	0.6
Blood loss (ml), mean ± SD	195 ± 126	160 ± 104	0.1
Operative time (min.), mean ± SD	160 ± 33	166 ± 41	0.3
RALP without PLD, no. (%)	33 (68.8%)	65 (54.6%)	0.09
RALP with PLD, no. (%)	15 (31.2%)	54 (45.4%)	0.09
Catheter time (days), mean ± SD	14.2 ± 2.1	14.3 ± 2.8	0.9

*PLD* Pelvic lymph node dissection, *RALP* robotic-assisted laparoscopic prostatectomy, *SD* standard deviation

Examination of the surgical specimens revealed no significant differences between the groups with respect to tumor stage and Gleason score, although a tendency toward more T3 and T4 tumors and Gleason grade ≥ 8 was observed in the suspension group (Table 3). The positive margin rate for T2 tumors was similar in both groups, but was statistically significant higher in the suspension group for T3 and T4 tumors. However, when a comparison of positive surgical margins of the prostatic apex was performed exclusively, the significant difference disappeared. There was no difference in biochemical recurrence between the groups.

**Table 3:** Postoperative outcomes

	Without suspension (*n* = 48)	With suspension (*n* = 119)	*p*
T stage (%)			
pT2	37/48 (77.1%)	76/119 (63.9%)	0.1
pT3/4	11/48 (22.9%)	43/119 (36.1%)	0.1
PSM rates (%)			
pT2	5/37 (13.5%)	11/76 (14.5%)	0.9
pT3/4	0/11 (0%)	15/43 (34.9%)	< 0.05
PSM at the apex (all)	5/48 (10.4%)	14/119 (11.8%)	0.8
PSM at the apex pT2	5/37 (13.5%)	4/76 (5.3%)	0.1
PSM at the apex pT3/4	0/11 (0%)	10/43 (23.3%)	0.1
BCR (%)			
T2	3/37 (8.1%)	3/76 (3.9%)	0.4
T3/4	2/11 (18.2%)	9/43 (20.9%)	0.8
Gleason score (%)			
3 + 3	9 (18.8%)	21 (17.6%)	0.9
3 + 4	23 (47.9%)	57 (47.9%)	1.0
4 + 3	12 (25%)	19 (16.0%)	0.2
≥ 8	4 (8.3%)	22 (18.5%)	0.1

*BCR* Biochemical recurrence, *PSM* positive surgical margins

Continence rates preoperatively, 3 months after RALP and 18 months after RALP, are displayed in Table 4. The preoperative data were unfortunately incomplete, with 46% of the forms from the study population absent. In patients from which EPIC-26 could be obtained, preoperative continence rate was 100% in the non-suspension group compared to 93.7% in the suspension stitch group (*p* = 0.4).

**Table 4:** Continence rate

Continence	Without suspension (*n* = 11)	With suspension (*n* = 79)	*p*
Preoperatively	11 (100%)	74 (93.7%)	0.4
	Without suspension (*n* = 48)	With suspension (*n* = 119)	
3 months	17 (35.4%)	73 (61.3%)	< 0.005
	Without suspension (*n* = 43)	With suspension (*n* = 107)	
18 months	35 (81.4%)	97 (90.7%)	0.1

Three months after surgery, the odds ratio of experiencing urinary leaks over the past 4 weeks was 2.1 times higher (95% CI 1.0–4.3) in the non-suspension stitch group compared to the suspension stitch group (*p* < 0.05). Urinary continence was 61.3% in the suspension stitch group compared to 35.4% in the figure-of-eight stitch group at 3 months following robotic prostatectomy (*p* < 0.005). In ordinal regression analysis, the suspension stitch, bilateral nerve-sparing and body mass index were found to be independent predictors of urinary continence at 3 months (Table 5). The odds ratio of being continent 3 months post-prostatectomy was 2.5 times higher (95% CI 1.3–4.9) in the suspension stitch group compared to the non-suspension stitch group (*p* < 0.01). For bilateral nerve-sparing, the odds ratio of retaining urinary continence 3 months after surgery was 3.0 times higher (95% CI 1.2–7.2) than for patients who had prostatectomy without nerve-sparing (*p* < 0.05). A positive effect of unilateral nerve-sparing as well as posterior reconstruction on urinary continence was also suggested from the analysis, but neither of these reached statistical significance in our model. An increase in body mass index was associated with a decrease in the odds of urinary continence recovery with an odds ratio of 0.90 (95% CI 0.82–0.98, *p* < 0.05). There was no association between urinary continence and age or urinary continence and prostate volume at 3 months after surgery.

**Table 5:** Odds ratios, 95% confidence intervals and *p* values for predictors of urinary continence recovery 3 months after robotic prostatectomy

Parameter	Odds ratio	95% CI	*p*
Age	1.0	0.95–1.06	1.0
Body mass index	0.9	0.82–0.98	< 0.05
Nerve-sparing, bilateral	3.0	1.22–7.21	< 0.05
Nerve-sparing, unilateral	1.4	0.70–2.90	0.3
Posterior reconstruction	1.5	0.74–3.14	0.3
Prostate volume	1.0	0.98–1.01	0.4
Suspension stitch	2.5	1.30–4.88	< 0.01

The EPIC-26 forms 18 months post-prostatectomy were only submitted anonymously by 50 patients (30%). The remaining 117 patients were contacted by phone, of which 100 responded, resulting in a response rate of 90%. Urinary continence rates 18 months after surgery were 90.7% in the suspension stitch group versus 81.4% in the non-suspension stitch group (*p* = 0.1).

## Discussion

At our institution, the figure-of-eight stitching of the DVC was gradually abandoned and replaced by the periurethral suspension stitch after 300 cases. This was done in order to determine whether introduction of this particular stitching technique would improve early continence, as previously demonstrated in a study by Patel et al. [8]. Between May 28, 2015, and July 13, 2017, patients who underwent RALP were subjected to either the standard figure-of-eight stitch or the periurethral suspension stitch, according to the surgeons’ preference. During this period, all four surgeons in the robotic program eventually changed their DVC stitching technique from the standard figure of eight to the retropubic stitching technique. As such, the study includes the learning curve for the suspension technique but not for the figure-of-eight stitch, which had been practiced in the 300 previous cases. Although it is not difficult to perform, we speculate that the effect of the suspension stitch would have been even more pronounced, had it already been implemented as a routine step before the first patient was included in the study.

The learning curve of the prostatectomy procedure for the surgeons involved should also be mentioned as a possible bias, since the majority of the cases in the non-suspension stitch group were performed in the beginning of the study period, while most of the cases with the suspension suture were done in the tail end. The average number of previously performed cases per surgeon was around 100, which is considerably lower than what is typically reported in studies from high-volume institutions. Although there is no standard definition of the learning curve of robotic prostatectomy, a requirement of between 100 and 300 cases has been suggested to obtain the so-called «satisfactory outcomes» [9]. For urinary continence, however, satisfactory outcomes were suggested by a group of high-volume surgeons to be achieved after 100 cases indeed, as compared to 200 and 300 for potency and surgical margins, respectively [9]. The lower volume needed to achieve satisfactory continence was explained with the vesico-urethral anastomosis considered an easier part of the procedure, in contrast to nerve-sparing and dissection. In order to analyze whether the learning curve was affecting the early continence data, the first 24 cases with the figure-of-eight stitch were compared to the last 24. Although the early continence rate for the 24 first cases in the figure-of-eight stitch group was somewhat lower than that of the last 24 (29.2% vs. 41.7%), no significant difference was found (*p* = 0.4). In the suspension stitch group, on the other hand, the early continence rate from the first 59 patients was almost indistinguishable from that of the last 60 patients (61.0% vs. 61.7%, *p* = 0.9). However, the effect of the learning curve and experience should not be overlooked in this study. Especially since these results are based on a small group of patients and from surgeons, who on the evidence of their previously reported cases, were most certainly still in the process of refining the surgical procedure.

Rocco et al. have previously published a systematic review, in which posterior reconstruction of the rhabdosphincter was reported to increase recovery of incontinence within the first 30 days after RALP [7] and also demonstrated an improvement on continence after 90 days utilizing this particular surgical technique [10]. A posterior reconstruction as described by Rocco et al. was performed in around 25% of the cases in the present study. Although a trend toward a beneficial effect on early continence was observed in our data, the difference in favor of posterior reconstruction was not statistically significant.

Nerve-sparing has previously been suggested to increase continence at 6 months due to preserved innervation of the rhabdosphincter [7] and as such represents a possible confounder in this study. Although the number of patients who were subjected to bilateral nerve-sparing was similar in the two groups, patients in the non-suspension group were more likely to have a prostatectomy without any preservation of the nerves. However, after adjusting for differences in nerve-sparing between the groups, the periurethral retropubic suspension stitch was still found to be an independent predictor of improved continence 3 months after RALP. Bilateral nerve-sparing was also identified as an independent predictor of early continence recovery. A trend toward a positive effect of unilateral nerve-sparing on early continence recovery may also be suggested from the analysis, but did not reach statistical significance in our model.

The periurethral suspension technique was originally developed for the open retropubic prostatectomy [11] and later introduced into the robotic setting by Patel et al. [8]. The hypothesis was that the suspension stitch would reconstruct the puboprostatic ligament and thus provide support for the striated sphincter. As suggested by Patel et al., the periurethral stitch may also lead to stabilization of the posterior urethra and facilitate preservation of urethral length during dissection of the prostatic apex. It also helps control the venous bleeding from the DVC, possibly leading to increased vision during apical dissection.

Another potential explanation to the differences in early continence between the groups in the present study may be related to the way in which the DVC was managed. The striated urethral sphincter is localized just behind the DVC, which makes it susceptible to injury if the suture is placed too deep [12, 13]. Anterolateral components of the neurovascular bundle may also be at risk, although the majority of the neurovascular bundle is localized in the posterolateral region [14, 15]. In the present study, stitching of the DVC was performed in both groups, i.e., the figure-of-eight stitch in the non-suspension group and an absorbable suture around the DVC once, followed by a suspension suture twice around the DVC and in the perichondrium of the pubic bone in the suspension stitch group. As such, a suture was placed three times around the DVC per procedure in the suspension stitch group compared to two times in the non-suspension stitch group, making the striated urethral sphincter more at risk in the suspension stitch group. However, the suturing around the DVC was not performed identically in the two different groups. As demonstrated in Figs. 1, 2, the puboprostatic ligament was divided more extensively in the suspension stitch group, leading to improved visual control of the DVC. This may in fact have resulted in fewer sutures injuring the urethral sphincter in the suspension stitch group, possibly contributing to the improved continence in this group.

Although the DVC was stitched in both groups in the present study, there was a tendency toward less blood loss in the suspension group with an average blood loss of 160 ml compared to 195 ml in the non-suspension group. The difference in blood loss was not statistically significant, but it is worth pointing out that there were actually more pelvic lymph node dissections in the suspension stitch group. It may therefore be suggested that a significant difference in favor of the suspension stitch group could have been detected, if the number of pelvic lymph node dissections had been more evenly distributed between the groups. However, whether less bleeding from the DVC in the suspension stitch group resulted in better visual control of the apical dissection, with improved preservation of urethral length as a consequence, remains speculation.

The continence rate at 18 months for all patients in this study was 88%, with a 90.7% 18-month continence rate in the suspension stitch group compared to 81.4% in the non-suspension stitch group. This difference was almost significant with a *p* value of 0.1. A similar trend was seen in the study by Patel et al., who reported an impressive 12-month continence rate of 98% in the suspension stitch group compared to 96% in the non-suspension stitch group [8]. However, our long-term continence data are less reliable than those after 3 months, since a substantial number of the patients failed to submit their EPIC-26 forms anonymously. The majority of these data are collected through phone call interviews, which are more likely to be affected by bias from either respondent or interviewer.

This study has several limitations. The design is retrospective, and the number of patients is relatively low. Moreover, a substantial number of continence data preoperatively and after 18 months were absent. However, for patients who succeeded in submitting information on early continence rate following surgery, our data suggest an effect of the periurethral suspension stitch.

In conclusion, the periurethral retropubic suspension stitch improved early continence following robotic prostatectomy compared to the standard dorsal venous complex stitch at our department. Our results comply with the results of previously reported studies.

## Electronic supplementary material

Below is the link to the electronic supplementary material.

Supplementary file1 (MP4 17349 kb)

Supplementary file2 (MP4 16402 kb)
